# Examining the acceptance of drone delivery services among Chinese consumers: A perspective from urban and rural areas

**DOI:** 10.1371/journal.pone.0333422

**Published:** 2025-09-29

**Authors:** Jingqiong Wu, Ziwei Chen, Zhixian Zhang, Mingrui Cen

**Affiliations:** School of Traffic Engineering, Kunming University of science and Technology, Kunming, China; King Fahd University of Petroleum & Minerals, SAUDI ARABIA

## Abstract

Drones hold considerable promise in the realms of logistics and distribution, as they are expected to enhance the speed and efficiency of courier deliveries while simultaneously reducing associated costs. This study investigates Chinese consumers’ willingness to accept drone delivery services in the context of urban–rural disparities, conducting a comparative analysis to identify similarities and differences in the influencing factors across residential settings. The Technology Acceptance Model and the Environmental Literacy Model were extended incrementally using an approach to more comprehensively capture the factors influencing consumers’ willingness to adopt drone delivery services. Based on these extended frameworks, nine research hypotheses were proposed and empirically tested through a questionnaire survey. Data collected from 498 respondents was analyzed using a ridge regression model. Furthermore, using an additional independent test set of 102 questionnaires, we confirmed that ridge regression outperformed multiple linear regression in reducing error metrics, thereby demonstrating superior predictive accuracy and robustness. The results reveal significant regional differences in factors influencing consumers’ willingness to accept drone delivery services. In urban areas, environmental cognition, perceived usefulness, perceived ease of use and social norm significantly contribute positively to acceptance levels while perceived risk has a significant negative impact. Furthermore, policy support, UAV-environmental cognition, health safety, and service performance exert a relatively minor yet notable positive influence on acceptance levels within urban settings. In contrast, for rural consumers, environmental cognition, perceived usefulness, and perceived ease of use were key positive factors. UAV-environmental cognition and service performance also emerged as major contributors, exerting particularly strong positive effects on acceptance. Perceived risk negatively affected acceptance but to a lesser extent than in urban areas, whereas health safety, policy support, and social norm show no significant impact. Given these pronounced urban–rural disparities, logistics providers and policymakers are advised to implement context-specific, differentiated strategies: urban areas should focus on risk mitigation, regulatory enhancement, and policy incentives, whereas rural areas would benefit from increased awareness campaigns, pilot demonstrations, and infrastructure investment. Such targeted approaches are essential for optimizing the adoption and societal benefits of drone delivery services across diverse consumer groups.

## 1. Introduction

**Fig 1:**
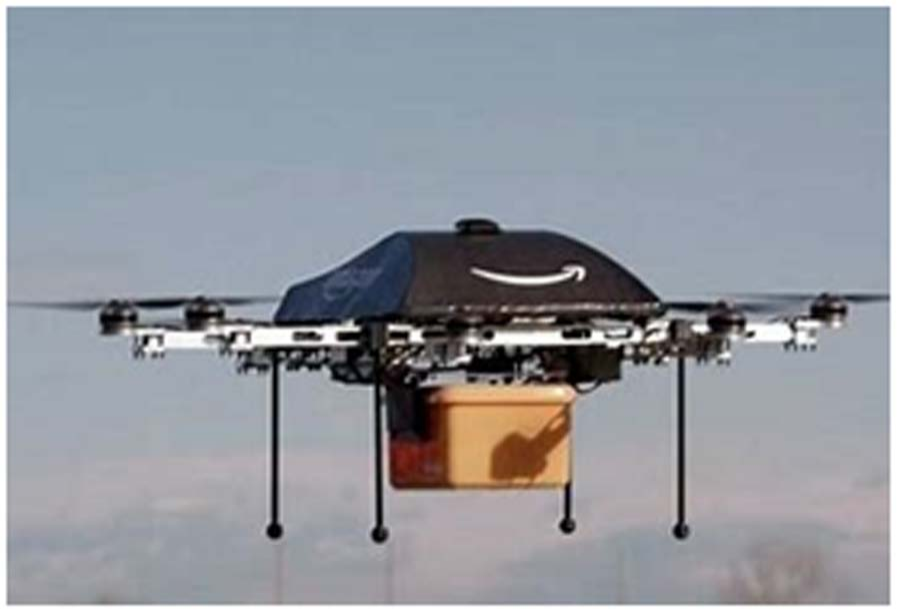
Amazon’s drone delivery program launch.

**Fig 2:**
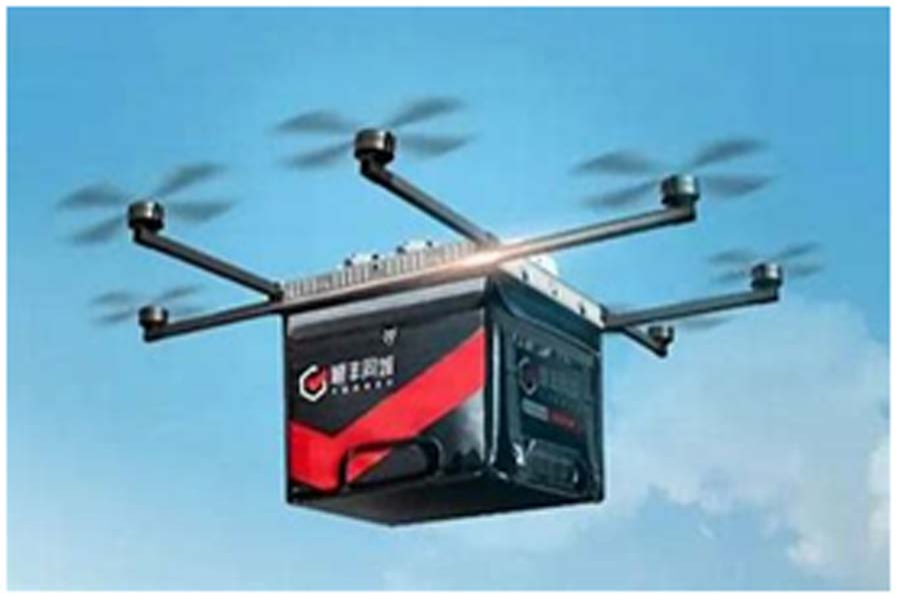
SF Express pilots drone deliver in Shenzhen.

With the continuous advancements in technologies such as artificial intelligence and automation, the efficient deployment of drones has become increasingly prevalent across a range of sectors, including military operations, security, and agriculture, as well as for identification and monitoring purposes. This trend underscores the immense developmental potential of drone technology. Furthermore, the rapid global expansion of e-commerce has driven a surge in consumer demand for online shopping and expedited delivery services, attracting considerable attention from logistics service providers toward drone-based delivery solutions. In response, numerous logistics companies have launched pilot projects in recent years to explore the integration of drones into their delivery systems. For example, Amazon has announced plans to commence drone deliveries for its Prime Air customers in California [1], aiming to enhance the efficiency of rapid delivery services (see Fig 1). Similarly, UPS has successfully conducted drone delivery trials in Florida and completed drone-based ice cream deliveries in New York [2]. In China, logistics companies such as SF Express (see Fig 2), as well as e-commerce platforms like JD.com and Meituan, have intensified their research and development efforts on drone technology and are actively advancing drone delivery services. Although these initiatives have predominantly focused on urban and suburban areas—where infrastructure is well-developed and consumer demand is concentrated—applications in rural regions are gradually emerging as a promising new frontier. For instance, JD.com has implemented drone delivery projects in several rural provinces of China [3]. Internationally, Zipline has established drone networks to deliver medical supplies to remote locations in countries such as Rwanda and Ghana [4].

Despite the growing enthusiasm for drone delivery, significant challenges remain, particularly regarding consumer acceptance. While drones offer notable advantages over traditional truck-based delivery, such as faster delivery, increased efficiency, lower costs, energy savings, and reduced environmental pollution [5], consumers continue to express concerns about potential drone malfunctions, privacy violations, and inaccurate deliveries [6]. These concerns are not uniform and may vary depending on regional contexts, highlighting the importance of examining drone acceptance from a geographic perspective.

Specifically, the introduction of drone delivery services in urban and rural areas has distinct implications that remain underexplored. Urban areas may experience faster adoption due to better infrastructure, higher technological readiness, and greater exposure to innovative delivery solutions [7]. However, urban consumers may also demonstrate heightened sensitivity to privacy infringements and airspace restrictions, complicating the seamless integration of drones into the existing delivery infrastructure [8,9]. Conversely, rural regions present fewer physical obstacles, such as traffic congestion, creating a potentially favorable operational environment for drones. Nevertheless, rural communities may face unique challenges such as concerns regarding service reliability, lower technological literacy, and cautious attitudes toward adopting new technologies due to limited exposure and perceived risks of technological failure [10,11]. Therefore, gaining a deeper understanding of how urban-rural differences influence consumer acceptance is critical for effectively deployment of drone delivery services. Building on these urban–rural differences, the purpose of this study is to address the following research questions (RQs).

RQ1: What factors may influence Chinese consumers’ willingness to adopt drone delivery services, and do these factors differ systematically between urban and rural populations?

RQ2: If systematic differences exist, which factors are more critical in urban contexts and which in rural contexts, and what implications how these differences hold for differentiated deployment strategies and policy design?

In response to RQ1–RQ2, this paper examines Chinese consumers’ willingness to adopt drone delivery services, with a particular focus on the contrasts between urban and rural settings. By incrementally extending the Technology Acceptance Model and the Environmental Literacy Model, this study develops a comprehensive analytical framework empirically validated through questionnaire surveys and rigorous statistical data analysis. This study systematically identifies and compares the key factors influencing the acceptance of drone delivery services among both urban and rural consumers. Furthermore, it offers targeted recommendations for logistics enterprises and policymakers to facilitate effective deployment of drone delivery services across different regions.

The originality of this study lies in its comparative perspective, as it integrates both urban and rural populations within a unified empirical framework. This approach addresses the limitations of previous technology acceptance research, which has predominantly focused on general trends while neglecting regional characteristics, urban–rural disparities, and insufficient representation of rural populations. In addition, this study contributes methodologically by applying ridge regression, which offers greater robustness to multicollinearity compared to traditional regression techniques. This is particularly important in urban–rural comparative research, where heterogeneous samples and overlapping explanatory variables often increase the risk of multicollinearity. By mitigating such risks, ridge regression not only enhances the reliability of the empirical results but also provides a valuable methodological reference for future research on technology acceptance and consumer behavior. Insights derived from this study will enable logistics providers and policymakers to formulate tailored deployment strategies, effectively addressing specific regional challenges and optimizing the benefits of drone delivery services for different consumer groups.

## 2. Literature review

Drone technology encompasses diverse systems and engineering disciplines, facilitating extensive applications across various sectors [12]. With its expanding range of uses, drones have been widely integrated into industries such as military operations, agriculture, and surveying. As their deployment continues to grow, drone technology has increasingly become a pivotal research field in numerous domains. One significant advantage of drones is their capability to operate effectively in environments that are either inaccessible to or prohibitively expensive for human labor, greatly improving operational efficiency and reducing associated costs [13].

In logistics, specifically, drones have become a prominent technological solution, enabling faster and more efficient deliveries. This is particularly advantageous in urban settings, where drones can navigate shorter and more direct routes without being hindered by traditional road infrastructures or traffic congestion, thereby substantially enhancing delivery efficiency. Recent research efforts have concentrated on improving drone endurance [14,15] and optimizing flight paths [16,17], which further enhance operational efficiency, reduce operational costs, and improve flight safety. In addition, studies have shown that integrated drone-truck operations hold promise for faster and more efficient overall delivery, while reducing the per-mile cost of delivery [18–20]. Beyond these technological advances, considerable attention has recently been directed toward the integration of intelligent scheduling systems and artificial intelligence algorithms to facilitate collaborative operations among multiple drones. Such integrations promise enhanced flexibility, efficiency, and intelligent control of logistics distribution [21,22]. Moreover, topics such as urban airspace management [23,24], regulatory adaptation [25], and environmental impacts of drones [26,27] have become increasingly prominent in drone-related research.

Despite the considerable potential of drones in logistics and delivery, existing research has primarily focused on technical dimensions, such as improving drone performance, optimizing routes, multimodal cooperative deliveries, intelligent scheduling systems, and airspace management. However, the critical aspect of user acceptance of drone-based delivery services has not received sufficient attention. User acceptance significantly influences not only the practical implementation of drone logistics but also policymaking, operational model refinement, and integration into social environments. To address this gap, this study provides an in-depth literature review of relevant scientific articles, with the aim of enhancing the understanding of the context and factors influencing user acceptance.

Consumer attitudes toward drone delivery vary significantly across countries and regions, with developing countries often showing more enthusiasm than developed countries. A recent international survey by McKinsey found that the willingness to use drone deliverie was above 80% in countries like China, India, Brazil, and Saudi Arabia, compared to roughly 50–60% in the United States and Germany [7]. Many consumers in emerging economies are open to drones as a leapfrogging technology for faster and more reliable logistics, while consumers in some developed markets appear more skeptical or comfortable with existing delivery systems. Recent literature and survey data shed light on the underlying reasons behind these disparities. In many developed countries, public concerns about drones are primarily focused on issues of safety, noise, and privacy—factors that often temper enthusiasm despite the potential advantages of drone technology. For example, a representative study in Germany found that anticipated risks—such as increased low-altitude air traffic, noise pollution, and visual disruption—significantly dampen public attitudes toward drone delivery, outweighing perceived benefits such as faster or more flexible logistics [28]. Similarly, surveys conducted in the United States reveal widespread caution: more than half of United States respondents reported little or no trust in drones to deliver products safely, citing fears of accidents and data privacy breaches as major concerns [29]. These findings suggest that in industrialized nations with established delivery infrastructures, perceptions of technological risk and lower tolerance for potential disturbances are critical barriers to public acceptance.

In contrast, developing countries and emerging economies tend to exhibit greater optimism toward drone delivery services, often viewing the technology as a means to address infrastructural shortcomings. Analysts have suggested that drone logistics enable such countries to leapfrog expensive traditional infrastructure, allowing goods to be delivered to remote or underserved areas without the need for extensive road networks. [30] This leapfrogging effect may partly explain the greater enthusiasm observed among consumers in countries such as India, Rwanda, and China, where drones offer tangible improvements in service quality in contexts where ground transportation is often limited or inefficient.

Within countries, urban-rural disparities also significantly influence consumer acceptance. Urban residents, who are more frequently exposed to app-based logistics services and emerging technologies, demonstrate higher levels of familiarity with and openness to drone delivery. According to Cornell et al. [7], 80% of respondents living in or near major cities expressed willingness to adopt drone delivery, compared to 67% in more remote areas. Although urban consumers may benefit from increased convenience, concerns persist about privacy, safety, and the risk of technical malfunctions or inaccurate deliveries [8,13,31–33]. Moreover, the high population density and regulatory complexity in urban environments necessitate cautious implementation by companies and policymakers.

In contrast, rural and remote regions are often viewed as favorable environments for piloting drone delivery systems due to lower population density, minimal airspace congestion, and limited access to conventional logistics. In such areas, drones can help bridge the “last-mile” delivery gap, particularly where infrastructure is underdeveloped or traditional services are inefficient [9]. In China, for instance, e-commerce firms such as JD.com have launched drone delivery initiatives in rural provinces to reduce costs and improve logistics efficiency [3,34]. These efforts have received governmental support as part of broader rural revitalization strategies. Real-world applications further reveal that acceptance is highest when drones fulfill critical local needs. In Rwanda and Ghana, for instance, drone networks operated by Zipline deliver life-saving medical supplies to isolated communities, effectively addressing chronic infrastructure gaps [4]. These services, endorsed by governments and integrated into healthcare systems, have helped normalized drones as beneficial tools, with high public approval due to their tangible impact. Ultimately, consumer acceptance is most pronounced in contexts where drone delivery demonstrably improves livelihoods and addresses critical service gaps. Although rural environments pose fewer logistical barriers for drone delivery, challenges such as ensuring service reliability, overcoming lower technological literacy, and addressing cautious attitudes toward new technologies still remain [6,10,35]. Indeed, studies indicate that rural communities’ hesitancy in embracing drone technology is largely due to their limited exposure to such innovations and concerns about potential technological failures [10,35]. These factors must be considered when assessing the full potential for drone delivery in rural settings.

Across the literature, several key factors emerge that influence whether consumers accept or reject drone-based delivery. One major driver is perceived usefulness or added value: Consumers are more likely to embrace delivery drones if they believe they offer clear advantages, such as faster delivery times, improved access (especially in hard-to-reach areas), or enhanced safety and convenience [11,33,36–43]. A global survey noted that by 2023, speed of delivery had become the most attractive aspect of drone services for consumers (overtaking cost considerations), with many willing to pay a premium for urgent deliveries via drone [7]. In China’s cities, early adopters have praised drones for their speed and reliability—for instance, food orders delivered by drone in Shenzhen arrive within seconds of the estimated time, and customers appreciate avoiding traffic delays and human contact for routine orders [44].

The COVID-19 pandemic further amplified this perception of usefulness, serving as a turning point in public acceptance. During the pandemic, drones were rapidly deployed in various countries to transport medical supplies, test kits, and even everyday goods to people under lockdown, highlighting their value in reducing human contact [36]. Studies examining this period showed a significant uptick in acceptance driven by health and safety motivations [36,42]. Yuen et al [42] found that fear of COVID-19 infection positively influenced people’s perceived utility of drone deliveries. In their survey of Singapore residents, those who felt more vulnerable to the virus and believed in drones’ efficacy as a contactless delivery method were far more likely to intend to use drone services. In fact, fear of contracting COVID-19 emerged as the single most influential factor increasing adoption intentions in that study. This suggests that in times of crisis, public priorities shift—concerns about privacy or noise fade into the background while the benefit of minimizing disease risk becomes paramount. Empirical evidence from China’s pandemic response reinforces how COVID-19 accelerated drone adoption. During citywide lockdowns, companies like JD.com and Antwork (Terra Drone) coordinated with local authorities to deliver necessities and medical samples via drones when conventional transport was restricted [45,46]. These drone flights, often publicized in the media, showcased the technology’s ability to operate in hazardous or inaccessible conditions, thereby improving public opinion. Community stakeholders who experienced drone deliveries for the first time during COVID-19 generally responded positively, associating drones with emergency relief and public benefit [47]. By proving their usefulness in a health crisis, drones overcame some of the skepticism—people saw them not just as a high-tech novelty, but as a practical solution to urgent problems. In summary, COVID-19 acted as a catalyst that both demonstrated the benefits of drone delivery and temporarily lowered the barriers to consumer acceptance, potentially leaving a lasting increase in willingness to use such services even beyond the pandemic context.

On the other hand, perceived risks and concerns significantly hinder acceptance. Privacy is a recurring concern in many studies [8,32,33], with consumers worrying about drones’ cameras or sensors collecting data, or simply feeling uneasy about unmanned devices near their homes. A survey of consumers in Pakistan (a developing country case) found that privacy issues were the primary concern regarding delivery drones, even more so than operational issues [32]. Safety and security concerns are also prominent. Urban residents, in particular, express fear of drones crashing into people or property. Until trust is built, many city dwellers are unwilling to have drones regularly flying overhead [31]. This hesitance is reinforced by the association of drones with military or surveillance uses in the public imagination, which can evoke anxiety [48].

Cost and economic factors also play a role. While companies claim that drones will eventually lower delivery costs, consumers may be skeptical if they are expected to pay a premium initially [2]. Some consumers are only willing to adopt drones if the service is cost-effective compared with existing options. However, evidence suggests that many consumers (about 58% globally) are willing to pay at least a small premium for the speed and novelty of drone delivery [7], indicating that cost may not be a prohibitive factor if other concerns (safety, privacy) are addressed.

Environmental and policy-related factors also influence drone delivery acceptance. From an environmental perspective, drone delivery has the potential to reduce carbon emissions and traffic congestion by replacing some truck or motorcycle deliveries. Studies [49] have conducted simulations suggesting that last-mile emissions could be reduced when drones are used optimally (for example, for lightweight, urgent deliveries). These sustainability benefits can improve public sentiment toward drones, especially as consumers become more environmentally conscious [35,50,51]. Some urban residents cite the eco-friendliness of drones—for example, their use of recyclable packaging and reduced fuel consumption—as a positive aspect [44]. However, environmental impacts can be a double-edged sword: while drones may reduce emissions, they also raise concerns such as noise pollution, visual disruption, and ecological disturbance, particularly in residential and natural areas [2,31,48]. Policy and regulation form the backbone of either enabling or hindering drone delivery, and by extension, shape public acceptance. In countries leading in drone deployment, regulators have begun crafting frameworks covering operator licensing, flight altitude restrictions, no-fly zones, and liability in the event of accidents [48]. For instance, the Civil Aviation Administration of China (CAAC) created a special license category for urban drone delivery, first issued in 2019 to support medical drone flights in Zhejiang [45]. Such regulatory support in China has been pivotal in legitimizing drone delivery trials across both rural and urban areas. On the other hand, in regions where regulations are lagging or overly strict, drone delivery projects often stall, limiting public exposure to the technology [7]. Many consumers have not seen or used drone delivery simply due to the lack of legal availability, which can breed uncertainty or indifference. Another policy aspect concerns how authorities address privacy and security legislation for drones—explicit rules against misuse (for surveillance or unsafe flying) can mitigate public fear. In summary, a supportive policy environment that prioritizes safety, privacy, and transparency can greatly enhance public acceptance, whereas regulatory vacuums or ambiguities tend to slow adoption by failing to address the public’s legitimate concerns [48].

Although research on drone delivery acceptance has expanded in recent years, important knowledge gaps remain—particularly in understanding urban-rural dynamics within developing countries:

Comparative Insights. Few studies provide a direct comparison of urban and rural consumer perceptions within the same cultural context. Most research to date has examined single-city samples or generalized national trends. This leaves a gap in understanding how urban-rural differences tangibly modify acceptance factors. A comparative study focusing on Chinese urban and rural consumers would shed light on whether factors like safety concerns or perceived benefits carry different weights in these settings.

Rural Consumer Perspectives. Rural populations are argely underrepresented in technology acceptance research for drone logistics. As one thesis notes, rural logistics needs and service disparities raise unique concerns about delivery equity and willingness to adopt drones [52]. Yet, empirical data on rural residents’ sentiments—beyond anecdotal evidence from pilot programs—remain scarce. Addressing this gap is crucial because assumptions drawn from urban-centric studies may not hold true in villages with different socio-economic conditions and technological exposure.

Long-Term Acceptance and Normalization. Another open question is how initial novelty effects or emergency-driven acceptance (e.g., during COVID-19) translate into sustained, routine use. Urban residents may have accepted drones for emergency needs, but will they embrace them for everyday parcel deliveries post-crisis? Longitudinal insights are needed to determine if high stated willingness will persist once drone deliveries become commonplace and the novelty has worn off.

Addressing these gaps is important for both theory and practice. By understanding the nuanced differences between urban and rural consumer acceptance in a country at the forefront of drone logistics such as China, researchers and practitioners can better tailor deployment strategies. Ultimately, filling these knowledge gaps will help ensure that drone delivery services are implemented in a ways that are sensitive to community concerns and that maximizes public benefit, thereby smoothing the path for broader adoption.

## 3. Theoretical modeling

### 3.1. Technology acceptance model

**Fig 3:**
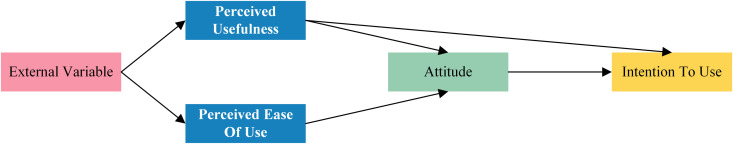
Technology acceptance model.

The Technology Acceptance Model (TAM) (Fig 3), developed by Davis [53,54], extends the theory of rational conduct and is primarily designed to explain and predict individuals’ willingness and behavior toward adopting new technologies. Davis emphasized that an individual’s acceptance of information technology is mainly influenced by factors such as perceived usefulness, perceived ease of use, and attitudes toward the technology. Perceived usefulness refers to the extent to which an individual believes new technology will enhance productivity, while perceived ease of use reflects the amount of effort an individual expects to be required to use the technology.

### 3.2. Environmental literacy model

**Fig 4:**
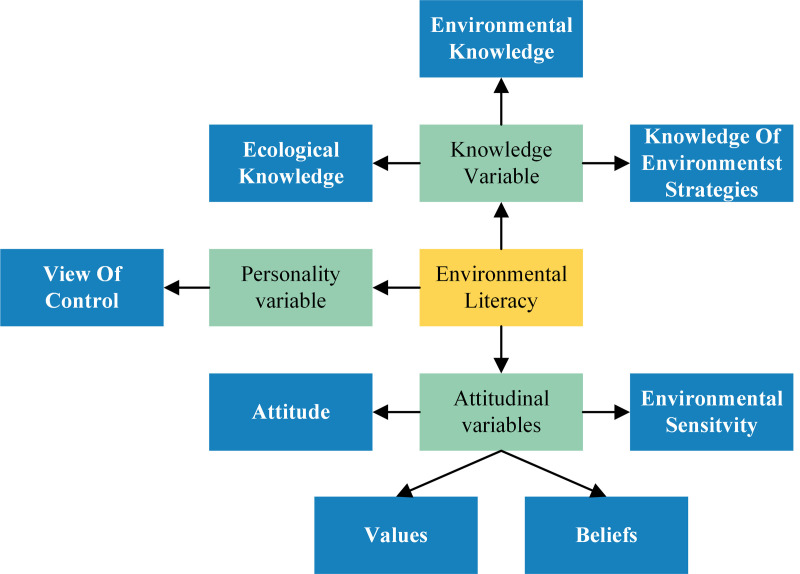
Environmental literacy model.

The Environmental Literacy Model (ELM) (Fig 4) was introduced by Sia, Hungerford, and Tomera [55] from a pedagogical standpoint while investigating the implementation of environmental behaviors among individuals. The model defines environmental behavioral intentions through the lens of environmental literacy, categorizing the environmental literacy of micro-subjects into three broad categories and eight dimensions. The three categories include knowledge, attitudinal, and personality variables. The eight dimensions are distributed as follows: three aspects under knowledge variables (environmental knowledge, ecological knowledge, and knowledge of environmental strategies); four aspects under attitudinal variables (attitudes, values, beliefs, and environmental sensitivity); and one aspect under the personality variable (view of control).

### 3.3. Selection of variables

The literature review highlights that the Technology Acceptance Model and the Environmental Literacy Model form the primary framework for understanding consumer acceptance of drone delivery. The key components of the technology acceptance model—perceived usefulness and perceived ease of use-are retained, while the two core elements of the environmental literacy model—environmental knowledge and environmental sensitivity-are incorporated as variables of environmental cognition (EC) and UAV-environmental cognition (UAV-EC) within the framework of drone delivery. However, due to the limitations of these two theoretical models, their expansion is required to address the research context more comprehensively during practical application. Based on an analysis of the existing literature, five additional factors have been incorporated into the original model: perceived risk (PR), health safety (HS), policy support (PS), social norms (SN), and service performance (SP). In total, ten factors have been identified as influencing consumers’ willingness to accept drone delivery: Environmental Cognition (EC), UAV-Environmental Cognition (UAV-EC), Perceived Usefulness (PU), Perceived Ease of Use (PEOU), Perceived Risk (PR), Health Safety (HS), Policy Support (PS), Social Norms (SN), Service Performance (SP), and the dependent variable Willingness to Accept (WA). These factors are classified into three categories: EC and UAV-EC under Knowledge Perception, PU, PEOU, and PS under Perceptive Factors, and HS, PS, and SN under Situational Factors.

### 3.4. Interpretation of variables and research hypotheses

#### 3.4.1. Knowledge perception.

Knowledge perception generally refers to the extent to which individuals understand the information and knowledge they possess. In this study, aligned with the concept of environmental literacy, two variables were examined: environmental cognition and UAV-environmental cognition, both classified as knowledge perception. Environmental cognition involves the awareness and understanding of ecological issues, which is used to gauge consumer concern about environmental matters [56]. Environmental cognition is defined as the level of concern and anxiety individuals exhibit toward environmental issues. UAV-environmental cognition is defined as the degree of consumer awareness of the ecological sustainability of drones. The specific research hypotheses are as follows:

Hypothesis 1a (H1a): Consumers’ environmental cognition increases their willingness to accept drone courier deliveries.

Hypothesis 1b (H1b): Consumers’ UAV-environmental cognition increases their willingness to accept drone courier deliveries.

#### 3.4.2. Perceptive factor.

Perceptive factors are various elements that shape how individuals interpret and understand information, objects, or environments. In this study, two components of the Technology Acceptance Model—perceived usefulness and perceived ease of use—were selected and classified as perceptive factors, taking perceived risk factors into consideration. “Perceived risk” refers to the sense of uncertainty or concern about a behavior and the seriousness or magnitude of its potential negative consequences [57]. Before utilizing a service, consumers form perceptions that may include considerations of risks related to security and privacy [58]. The specific research hypotheses are as follows:

Hypothesis 2a (H2a): Perceived usefulness increases the willingness to accept drone courier deliveries.

Hypothesis 2b (H2b): Perceived ease of use increases the willingness to accept drone courier deliveries.

Hypothesis 2c (H2c): Perceived risk negatively impacts the willingness to accept drone courier deliveries.

#### 3.4.3. Situational factors.

Situational factors in consumer behavior refer to the contexts and conditions that influence customer decisions and actions [59]. According to Belk [60], situational variables can significantly enhance the understanding and explanation of customer behavior. This study identifies and categorizes three situational factors: health safety, policy support, and social norms. Health safety refers to the protocols, standards, and procedures designed to protect employee health and maintain a safe working environment. Policy support encompasses a wide range of legislative, administrative, and economic measures implemented by the government to promote the development of a specific business or sector. Social norms refer to individuals’ perceptions of the prevalence of certain behaviors within their social environment, as well as their understanding of the expectations placed upon them by others in that context [61,62]. The specific research hypotheses are as follows:

Hypothesis 3a (H3a): Health safety increases the willingness to accept drone courier deliveries.

Hypothesis 3b (H3b): Policy support increases the willingness to accept drone courier deliveries.

Hypothesis 3c (H3c): Social norms increase the willingness to accept drone courier deliveries.

#### 3.4.4. Service performance.

Service performance refers to a service provider’s ability to meet established standards and satisfy customer expectations in service delivery [63]. It includes various dimensions such as efficiency, effectiveness, reliability, and customer satisfaction with the service. The specific research hypothesis is stated as follows:

Hypothesis 4 (H4): Service performance enhances the willingness to accept drone courier deliveries.

### 3.5. Research framework

**Fig 5:**
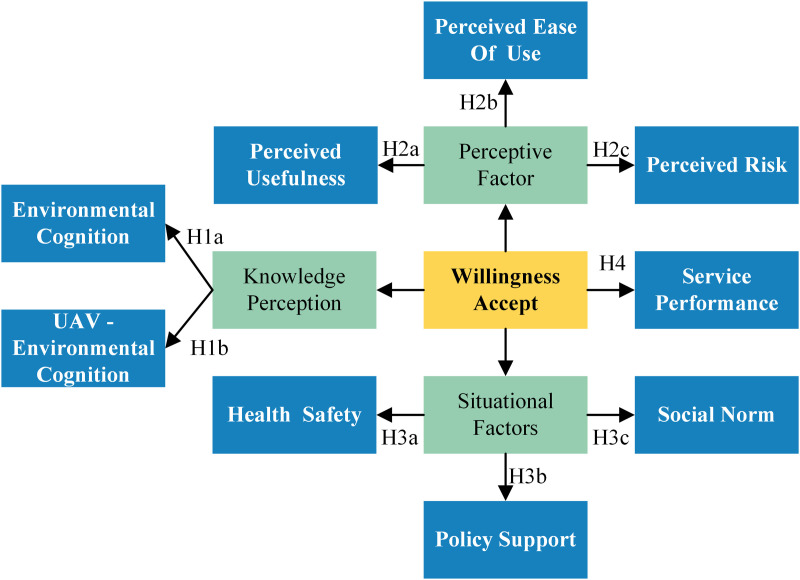
The particular research framework of this study.

By utilizing the Technology Acceptance Model and Environmental Literacy Model, and integrating insights from the current literature to identify and classify relevant factors, several research hypotheses are proposed. The specific research framework is illustrated in Fig 5. This framework contributes to the literature by providing a holistic analytical perspective that better reveals the mechanisms of consumer acceptance with respect to urban–rural differences in emerging economies.

## 4. Research subjects and methodologies

### 4.1. Subjects of study

For each construct, 3–5 items were selected. Some items were adapted from established Technology Acceptance and Environmental Literacy scales, others were drawn from prior literature, and several were newly developed to capture the unique context of drone delivery and the realities of urban-rural China. All items were measured on a 5-point Likert scale ranging from “strongly disagree” to “strongly agree,” which was chosen for its conciseness and adaptability. Given that the constructs were specified as reflective, Cronbach’s α was reported at the factor level. To enhance measurement transparency, item-level statistics such as communalities (extracted values) are presented in Appendix S1 Table, together with the full item list and citation sources.

In addition to construct measures, the questionnaire included demographic questions concerning respondents’ gender, age, education, current place of residence, occupation, and frequency of online shopping. To enhance representativeness, both online and offline distribution methods were used. The online survey reached respondents from various cities nationwide, ensuring high representativeness, while the offline survey targeted specific groups, providing a more focused sample. The combination of both methods enhances the efficiency of the survey and contributes to more accurate and comprehensive data. To accurately distinguish between urban and rural respondents, the study follows China’s official criteria for urban-rural classification: urban respondents are those residing in cities or towns, while rural respondents live in villages. The questionnaire categorized respondents’ areas of residence accordingly, ensuring a clear distinction between urban and rural populations.

The online questionnaire was distributed through the “Questionnaire Star” platform, while the offline questionnaire was administered in Kunming’s downtown business districts, university campuses, libraries, and subway stations for urban respondents. For rural respondents, survey points were selected from villages in Yuxi City, Dali Prefecture, Liupanshui City, and Honghe Prefecture—all representative of remote mountainous regions. These inland rural locations were chosen because they exhibit typical characteristics of less-developed areas in southwestern China, such as lower infrastructure density, limited market accessibility, and distinctive socio-cultural contexts. Coastal rural areas were not included because coastal cities are relatively more developed, and the rural–urban gap there is narrower, making them less representative of the inland rural realities targeted in this study.

In total, 350 online questionnaires were collected, of which 302 were valid, and 211 offline questionnaires were collected, with 196 being valid. These 498 valid questionnaires were used as the training set for fitting the regression models. Additionally, an extra set of 102 valid questionnaires was collected and designated as the test set to further evaluate the generalizability and predictive performance of the models. The inclusion of a separate test set allows for an unbiased assessment of model performance on unseen data, thereby providing a more robust comparison between ridge regression and multiple linear regression, while preventing overfitting to the training data.

### 4.2. Research methodologies

The data were analyzed using IBM SPSS Statistics 26 and ridge regression procedure from the predictive modeling module in SPSS Pro. This study employed several analytical techniques, including Cronbach’s alpha, exploratory factor analysis, Pearson correlation analysis, multiple linear regression analysis, and ridge regression analysis.

Multiple factors influence the willingness to accept drone delivery, and these factors are closely correlated. Using ordinary linear regression methods to analyze such data can often increase model error, reduce robustness, and result in inaccurate parameter estimation, potentially leading to incorrect conclusions.

Ridge regression, a biased estimation technique, is used to address covariance issues, particularly multicollinearity, by incorporating an L2 regularization term (the penalty term based on the sum of the squared coefficients) into the loss function. This method reduces the impact of multicollinearity on the model, improving both prediction accuracy and stability. Moreover, the penalty term helps eliminate extraneous noise, leading to a more streamlined and interpretable model [64]. Thus, this study constructs an indicator system for the factors influencing acceptance willingness and applies ridge regression analysis to statistically assess the determinants of willingness to accept for drone express delivery.

Ridge regression was chosen for several reasons: First, there was substantial multicollinearity among the independent variables, as indicated by high correlation coefficients and variance inflation factors in our preliminary analysis. Ridge regression effectively addresses this issue and improves the reliability of the results. Second, compared with other methods such as logistic regression, structural equation modeling (SEM), LASSO, and PLS-SEM, ridge regression was more suitable due to the relatively straightforward relationships between the variables in our model and the continuous nature of the dependent variable, willingness to accept. Other methods like logistic regression or SEM are better suited for different model structures, while LASSO and PLS-SEM are typically used for more complex, multi-layered models, which were not required for this study.

In addition, to demonstrate the advantages of ridge regression over linear regression in this study, we introduced several error metrics, including MAE (Mean Absolute Error), RMSE (Root Mean Square Error), and MAPE (Mean Absolute Percentage Error). MAE is suitable for measuring the overall average error and reflects the general accuracy of the model’s predictions. RMSE is more sensitive to large errors, thereby emphasizing the impact of extreme deviations. MAPE intuitively reflects the magnitude of model error relative to the actual values.

The predicted values for the test set were calculated using the fitted models, and the MAE, RMSE, and MAPE were computed separately for both linear regression and ridge regression on the same test set. This approach enabled a comprehensive evaluation of the predictive performance and robustness of the two models.

## 5. Data analysis

### 5.1. Dependability and validity

#### 5.1.1. Dependability assessment.

Cronbach’s alpha is a reliability measure used to evaluate the internal consistency of constructs. The overall Cronbach’s alpha coefficients for this study are presented in Table 1. The Cronbach’s alpha coefficients for all variables exceed 0.80, demonstrating that the questionnaire items in this study possess strong internal consistency.

**Table 1:** Cronbachs’ alpha coefficients. Cronbach’s α values ≥ 0.70 are considered acceptable, and values ≥ 0.80 indicate good reliability.

variant	Number of questions	Cronbachs’α
Environmental Cognition	4	0.879
UAV-Environmental Cognition	3	0.867
Perceived Usefulness	4	0.908
Perceived Ease Of Use	4	0.881
Perceived Risk	3	0.879
Health Safety	4	0.903
Policy Support	4	0.890
Social Norm	3	0.845
Service Performance	5	0.898
Willingness Accept	4	0.901

#### 5.1.2. Assessment of validity.

The literature suggests that Exploratory Factor Analysis (EFA) was employed to evaluate the questionnaire’s structural validity. The KMO sampling adequacy measure and Bartlett’s test of sphericity were used to assess the correlations among variables. Table 2 presents the KMO values for each variable along with the results of Bartlett’s test of sphericity.

**Table 2:** KMO values and Bartlett’s sphericity test. KMO values ≥ 0.70 indicate good sampling adequacy. A significant Bartlett’s test of sphericity (p < 0.001) confirms that the data are suitable for factor analysis.

Variant	KMO value	Bartlett’s spherical test	
		Approximate chi-square	df
Environmental Cognition	0.832	1022.493	6
UAV-Environmental Cognition	0.740	721.946	3
Perceived Usefulness	0.855	1299.073	6
Perceived Ease Of Use	0.838	1030.998	6
Perceived Risk	0.743	791.137	3
Health Safety	0.851	1236.732	6
Policy Support	0.838	1126.108	6
Social Norm	0.730	618.497	3
Service Performance	0.889	1397.464	10
Willingness Accept	0.847	1217.557	6

As can be seen from the table, the KMO values of the variables are all greater than 0.70, and the approximate chi-square is also more than adequate, satisfying the conditions for factor analysis. In addition, all Sig. values are 0.000, indicating that the assumption of sphericity was met.

### 5.2. Descriptive statistics

Table 3 presents the basic demographic statistics of the respondents. The sample includes 45.4% males and 54.6% females. Individuals aged 18–24 account for 32.5% of the respondents, representing Generation Z, a group known for its innovation and technological proficiency. This demographic is expected to play a pivotal role in driving the growth of drone logistics [65]. Individuals aged 25–34 make up 32.5% of the respondents, while those aged 35–44 account for 21.5%, indicating that the sample was predominantly young and representative of the demographics with the greatest market potential for drone delivery technology [66]. Consequently, the youthful demographic was disproportionately represented, with 86.5% of the sample aged between 18 and 44. Additionally, 71.9% of respondents reported a university education or higher, while 63.7% resided in urban areas and 36.3% in rural areas. In terms of online shopping behavior, 38.8% of participants shopped online an average of 5–7 times per month, while 32.9% made purchases 2–4 times per month. Furthermore, to assess potential sampling bias, the educational attainment distribution of the rural sample was compared with the National Bureau of Statistics’ 2023 rural education data. Results indicate that the rural respondents in this study have a relatively higher proportion of college-educated individuals than the national rural average, which should be taken into account when interpreting the findings.

**Table 3:** Results of basic personal statistics of respondents. Percentages may not sum to 100% due to rounding.

Basic personal statistics of respondents		n	%
Sexes	Male	226	45.4
	Female	272	54.6
Age	18-24 years	162	32.5
	25-34 years	162	32.5
	35-44 years	107	21.5
	45-54 years	49	9.8
	55-64 years	14	2.8
	65 years and over	4	0.8
Education attainment	Junior high school and below	51	10.2
	High School (Secondary)	89	17.9
	University (post-secondary)	289	58.0
	Graduate students and above	69	13.9
Current place of residence	Urban	317	63.7
	Rural	181	36.3
Type of occupation	Students	154	30.9
	Professional and technical staff (teachers, doctors, lawyers, etc.)	40	8.0
	Staff of government agencies and public institutions	69	13.9
	Enterprise personnel	117	23.5
	A private firm (PRC usage)	28	5.6
	Logistics industry practitioners	22	4.4
	Else	68	13.7
Frequency of online shopping (average times per month)	1 times or less	26	5.2
	2-4 times	164	32.9
	5-7 times	193	38.8
	8-10 times	80	16.1
	More than 10 times	35	7.0

### 5.3. Pearson correlation analysis

**Fig 6:**
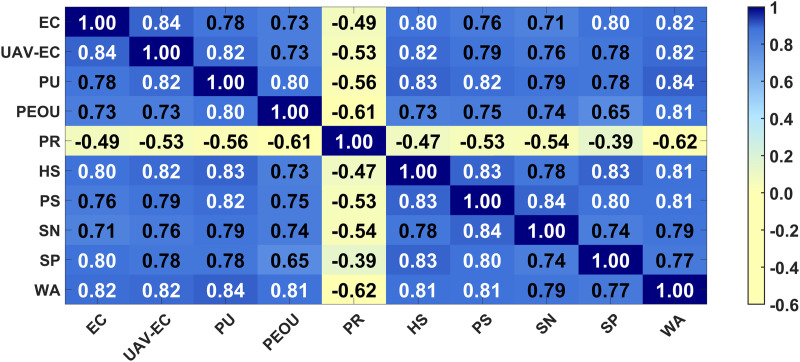
Pearson Correlation Matrix Heatmap. Correlation coefficients between 0.60 and 0.80 are generally considered to indicate a strong correlation, while values above 0.80 suggest a very strong correlation. Abbreviations: EC = Environmental Cognition; UAV-EC = UAV-Environmental Cognition; PU = Perceived Usefulness; PEOU = Perceived Ease of Use; PR = Perceived Risk; HS = Health Safety; PS = Policy Support; SN = Social Norms; SP = Service Performance; WA = Willingness to Accept.

Pearson correlation analysis assesses both the strength and direction of the relationship between two variables. A significant p-value of 0.0000 (p < 0.05) indicates a correlation between the variables. Fig 6 displays the correlation coefficient 𝑟 as a heat map, using color gradients to represent the magnitude of the values. The absolute value of the correlation coefficient between perceived risk and willingness to accept exceeds 0.60, while the correlation coefficients between the other variables and the dependent variable, willingness to accept, exceed 0.70. This indicates a strong linear relationship between the independent and dependent variables, making regression analysis appropriate. However, correlation coefficients among some independent variables exceed 0.80, suggesting potential multicollinearity, which could lead to suboptimal model fit and increased prediction error in the regression analysis.

### 5.4. Multicollinearity diagnostic results

The least squares method was applied for the initial multiple linear regression analysis, followed by an assessment of multicollinearity. Table 4 presents the analysis of variance, while Table 5 provides the regression parameter calculations and the multicollinearity assessment. The outcomes of the model evaluation are: R^2 ^= 0.828 indicates that 82.8% of the variability is accounted for by the model. The variance result shows F = 260.256, with a significance level of P < 0.0001, signifying an exceptionally significant regression effect and a high overall goodness-of-fit for the model. However, the preliminary Pearson correlation analysis indicates the presence of multicollinearity within the regression equation. The results of the Pearson correlation analysis initially confirm multicollinearity, as the variance inflation factor (VIF) for three explanatory variables exceeds 5, further reinforcing this issue. Additionally, the eigenvalues show more than one value approaching zero, and the condition indices exceed 10, signaling severe collinearity among variables. The multicollinearity among the explanatory variables can be mitigated through ridge regression analysis.

**Table 4:** Analysis of variance. This table reports the ANOVA results of the multiple linear regression model. The regression model is highly significant (F = 260.256, p < 0.0001), indicating strong overall explanatory power.

Model	Sum of squares	df	Mean square	F	Sig
Regression	354.571	9	39.397	260.256	0.000^b^
Residual	73.872	488	0.151		
Total	428.443	497			

**Table 5:** Regression parameter estimation and diagnosis of multicollinearity. This table presents the unstandardized and standardized regression coefficients, significance tests, and multicollinearity diagnostics. Several variables show variance inflation factors (VIFs) greater than 5, eigenvalues close to zero, and condition indices above 10, confirming the presence of multicollinearity.

Model	Unstandardized Coefficients		Standardized Coefficients	t	Sig.	Collinearity Statistics		
	B	Std.Error			Beta	VIF	Eigenvalues	Condition Index
Constant Term	0.62	0.142		4.36	^***^0.000		9.65	1.00
EC	0.19	0.04	0.18	4.56	^***^0.000	4.56	0.25	6.18
UAV-EC	0.11	0.04	0.12	2.84	^**^0.005	4.88	0.02	20.54
PU	0.15	0.04	0.16	3.63	^***^0.000	5.48	0.02	22.93
PEOU	0.16	0.03	0.16	4.50	^***^0.000	3.65	0.01	27.45
PR	−0.12	0.02	−0.14	−5.55	^***^0.000	1.75	0.01	29.31
HS	0.06	0.04	0.06	1.39	0.166	5.56	0.01	31.06
PS	0.05	0.04	0.05	1.21	0.228	5.38	0.01	34.06
SN	0.10	0.04	0.10	2.50	^**^0.013	4.08	0.01	35.61
SP	0.09	0.04	0.08	2.09	^**^0.037	4.57	0.01	37.99

### 5.5. Ridge regression analysis and hypothesis testing

**Fig 7:**
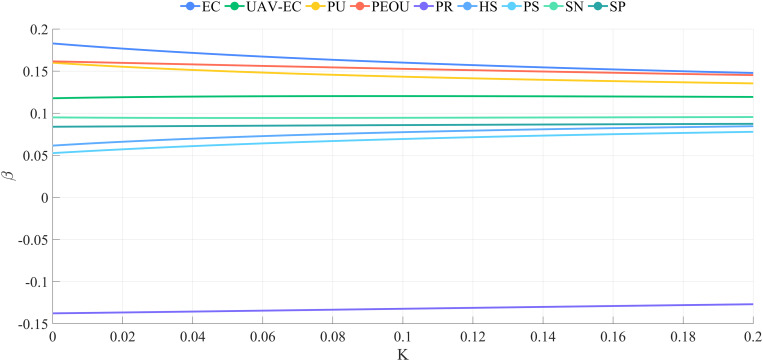
Ridge trace map. The plot shows that regression coefficients stabilize when k > 0.18, confirming that ridge regression effectively mitigates multicollinearity.

**Fig 8:**
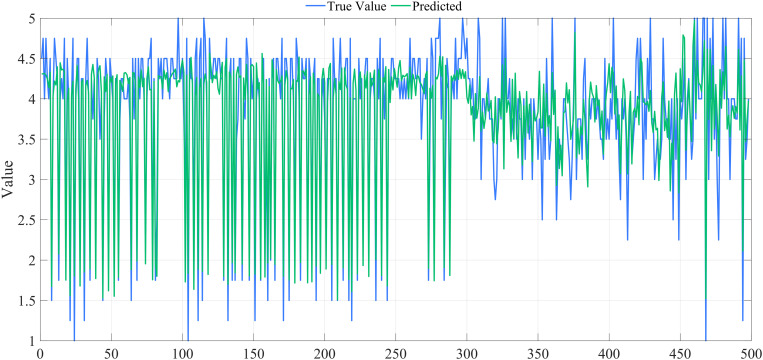
Figure of Ridge Regression Model Results. The scatter plot demonstrates a close alignment between predicted and actual values, with R² = 0.826, highlighting the strong explanatory power and predictive accuracy of the ridge regression model.

Ridge regression analysis was conducted using SPSS Pro to mitigate multicollinearity among explanatory variables and to evaluate the nine hypotheses suggested above. Fig 7 displays the ridge trace plot, illustrating that the ridge regression coefficients stabilize when k > 0.180. The overall fit of the ridge regression model is satisfactory, as shown in Fig 8.

For k = 0.183, R² = 0.826, thus k = 0.183 is suitable for ridge regression. This value of k was selected because the ridge trace plot indicated that the regression coefficients became stable when k exceeded approximately 0.180, and further increases in k did not lead to substantial changes in coefficients while also avoiding over-penalization. Selecting k = 0.183 therefore balances bias and variance, ensuring model stability and optimal predictive performance. The p-value of the F-test is 0.000, indicating a significant rejection of the null hypothesis, which confirms a regression relationship between the independent and dependent variables. The standardized ridge regression equation for willingness to accept based on the nine explanatory variables is shown in Eq. 1. In this equation, EC denotes environmental cognition, UAV-EC denotes UAV–environmental cognition, PU denotes perceived usefulness, PEOU denotes perceived ease of use, PR denotes perceived risk, HS denotes health safety, PS denotes policy support, SN denotes social norms, and SP denotes service performance. WA is the dependent variable, willingness to accept drone delivery.


$$\begin{array}{c} WA=0.674+0.153EC+0.112UAV\text{---}EC+0.131PU+0.146PEOU\\ -\;0.109PR+0.081HS+0.077PS+0.096SN+0.093SP \end{array}$$ (1)

All independent variables had a statistically significant effect (p < 0.001) on willingness to accept, highlighting the model’s coherence and explanatory strength in variable selection. Environmental cognition (Beta = 0.149), UAV-environment cognition (Beta = 0.120), perceived usefulness (Beta = 0.137), and perceived ease of use (Beta = 0.146) exerted significant positive influences on willingness to accept, while perceived risk (Beta = −0.128) had a significant negative effect. These results support hypotheses H1a, H1b, H2a, H2b, and H2c.

Additionally, social norms (Beta = 0.095), service performance (Beta = 0.087), health safety (Beta = 0.084), and policy support (Beta = 0.077) significantly and positively influenced willingness to accept, though their effects were relatively modest. Hypotheses H3c, H4, H3a, and H3b are empirically supported.

Overall, the findings confirm that all hypotheses from H1a to H4 are validated, with each variable exerting a significant, albeit varying, influence on the dependent variable, willingness to accept.

### 5.6. Comparative analysis of urban and rural groups

The sample was divided into two groups based on residential area to compare the effects of the independent variables: (1) the urban group and (2) the rural group. The urban group includes participants living in cities and towns, while the rural group consists of individuals residing in villages.

**Fig 9:**
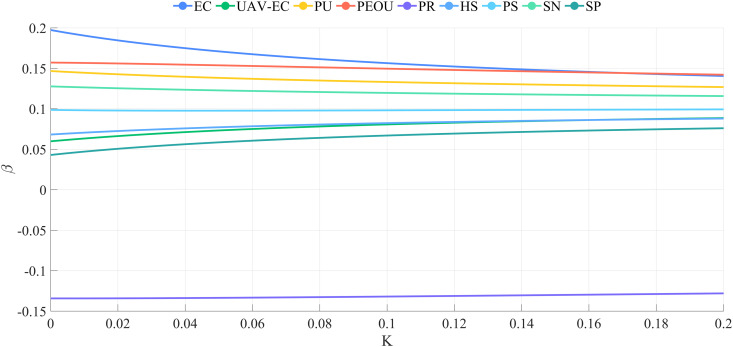
Ridge trace plot for the urban group.

**Fig 10:**
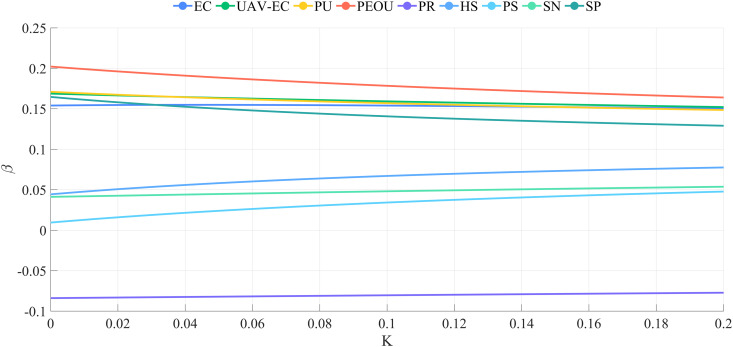
Ridge trace plot for the rural group.

Ridge regression analysis was conducted for both groups. Fig 9 displays the ridge trace plot for the urban group, indicating that the ridge regression coefficients stabilized until k > 0.180. When k = 0.182, R^2 ^= 0.873, suggesting that k = 0.182 is appropriate for ridge regression. Fig 10 presents the ridge trace plot for the rural group, where R^2 ^= 0.707 when k = 0.180, suggesting a weaker explanatory power compared with the urban group. These plots illustrate how ridge regression effectively addresses multicollinearity while highlighting differences in model fit between the two groups.

**Fig 11:**
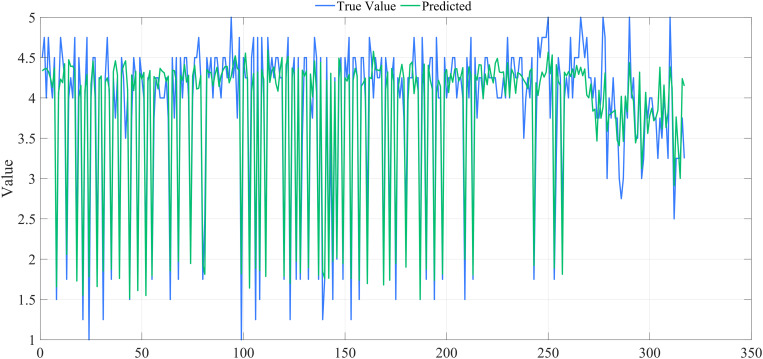
Figure of ridge regression model results for the urban group.

**Fig 12:**
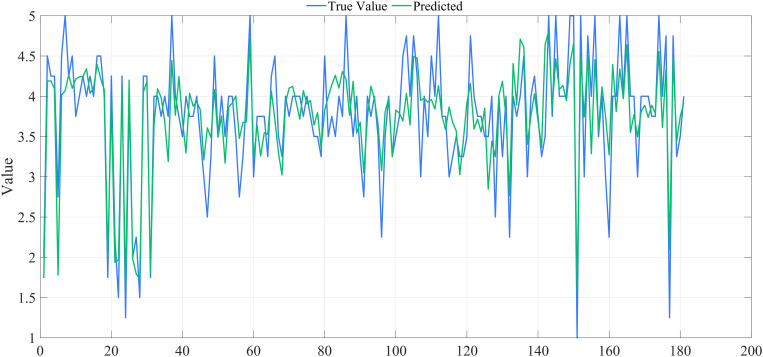
Figure of ridge regression model results for the rural group.

The overall fit of the ridge regression models was satisfactory for both groups, with a better performance observed for the urban group (Fig 11) and a reasonable fit for the rural group (Fig 12), where the predictive accuracy was somewhat lower compared with the urban group. These results demonstrate that ridge regression is effective in both contexts, but prediction is more challenging for the rural sample due to data characteristics.

**Fig 13:**
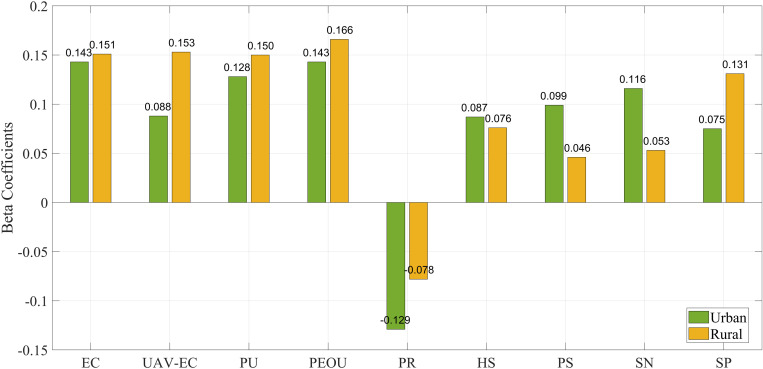
Comparison of beta coefficients for influencing factors between urban and rural groups.

Fig 13 visually illustrates the significant differences in the impact of these factors on willingness to accept between urban and rural groups, with the disparities in UAV-environmental cognition, perceived risk, policy support, social norm, and service performance being particularly pronounced between the two groups.

In the urban group, environmental cognition (Beta = 0.143), perceived usefulness (Beta = 0.128), perceived ease of use (Beta = 0.143), and social norms (Beta = 0.116) had a significant positive influence on willingness to accept, while perceived risk (Beta = −0.129) demonstrated a significant negative impact. Additionally, policy support (Beta = 0.099), UAV-environment cognition (Beta = 0.088), health safety (Beta = 0.087), and service performance (Beta = 0.075, p = 0.001) also exerted a favorable influence on willingness to accept, though their effects were relatively modest.

In the rural group, environmental cognition (Beta = 0.151), perceived usefulness (Beta = 0.150), and perceived ease of use (Beta = 0.166) had a significant positive impact. UAV-environment cognition (Beta = 0.153) and service performance (Beta = 0.151) also significantly influenced willingness to accept, more strongly than in the urban group. Perceived risk (Beta = −0.078, p = 0.035) had a notable negative effect on willingness to accept, though with a lesser impact compared to the urban group. Health safety (Beta = 0.076, p = 0.090), policy support (Beta = 0.046, p = 0.306), and social norms (Beta = 0.053, p = 0.222) had no significant influence on willingness to accept, a pattern not observed in the urban group. As summarized in Table 6, most hypotheses were supported in the urban group, while in the rural group, all hypotheses except H3a–H3c (health safety, policy support, and social norms) were supported.

**Table 6:** Summary of Hypotheses Testing Results for Urban and Rural Groups. Table summarizes hypothesis testing results for both urban and rural groups, indicating whether each hypothesis (H1a–H4) was supported.

Constant Term	Hypothesis	Urban group		Rural group	
		P	Supported (Yes/No)	P	Supported (Yes/No)
EC	H1a	^***^0.000	Yes	^***^0.001	Yes
UAV-EC	H1b	^***^0.000	Yes	^***^0.001	Yes
PU	H2a	^***^0.000	Yes	^***^0.001	Yes
PEOU	H2b	^***^0.000	Yes	^***^0.000	Yes
PR	H2c	^***^0.000	Yes	^*^0.035	Yes
HS	H3a	^***^0.000	Yes	0.090	No
PS	H3b	^***^0.000	Yes	0.306	No
SN	H3c	^***^0.000	Yes	0.222	No
SP	H4	^***^0.001	Yes	^**^0.003	Yes

### 5.7. Model Performance Comparison Test

**Fig 14:**
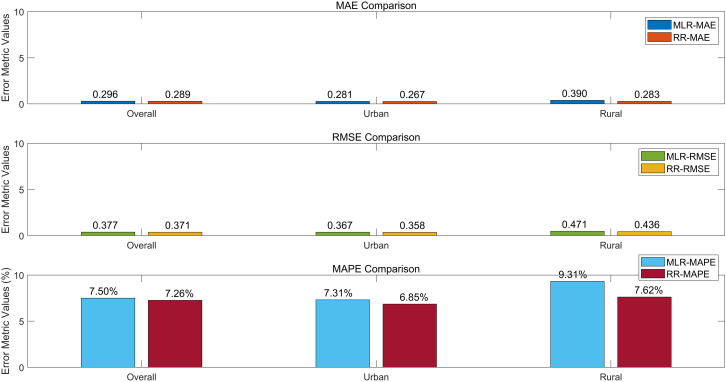
Test performance of multiple linear regression and ridge regression. Abbreviations: MLR = Multiple Linear Regression; RR = Ridge Regression; MAE = Mean Absolute Error; RMSE = Root Mean Square Error; MAPE = Mean Absolute Percentage Error.

The 102 valid questionnaires in the test set were divided into three groups: the overall group, the urban group, and the rural group. Models trained on the 498 questionnaires in the training set were used to predict the outcomes for each group in the test set. The MAE, RMSE, and MAPE values were then calculated for both the multiple linear regression and ridge regression models within each group. A visual comparison of the results is presented in Fig 14.

Compared with linear regression, ridge regression demonstrated improved performance on key error metrics across all three test datasets: the overall group, the urban group, and the rural group. For the overall group, MAE, RMSE, and MAPE were reduced by 2.36%, 1.59%, and 3.20%, respectively. In the urban group, the reductions were 4.98% for MAE, 2.45% for RMSE, and 6.29% for MAPE. In the rural group, MAE, RMSE, and MAPE decreased by 27.40%, 7.40%, and 18.20%, respectively.

These results highlight the value of ridge regression in addressing multicollinearity and improving predictive accuracy, especially in the rural dataset where variability is higher. Fig 14 thus demonstrates not only the robustness of ridge regression relative to traditional linear regression but also its contribution to enhancing the generalizability of the study’s findings across different population contexts. This provides methodological support for future acceptance studies and practical guidance for analyzing consumer behavior in diverse urban–rural settings.

## 6. Discussion

The results indicate that environmental cognition, UAV-environment cognition, perceived usefulness, perceived ease of use, and perceived risk are the key factors affecting consumers’ acceptance of drone delivery services. While these core factors are consistent with prior studies, the differences between urban and rural groups highlight the need for a more nuanced understanding of technology adoption in different environments.

Environmental cognition plays a crucial role in influencing technology acceptance, in both urban and rural areas. This indicates that, compared to the past, rural residents have become more aware of environmental issues. Notably, UAV-environment cognition has a more significant impact on acceptance in rural areas than in urban areas. This could be due to rural residents value drones as an eco-friendly alternatives to traditional delivery methods. In contrast, urban residents may focus more on the dual environmental impacts of drones, particularly concerns about noise and visual pollution, especially when drone traffic is frequent in quiet neighborhoods [2,31,48]. This discrepancy highlights regional variations in consumer concern for environmental issues. It should be noted, however, that the relatively high influence of environmental cognition and UAV-environment cognition in rural areas may also stem from potential sampling bias: the proportion of college-educated rural respondents in this study is higher than the national rural average.

Perceived usefulness and perceived ease of use are critical determinants of acceptance, which aligns with the conclusions of most previous studies [11,33,35,39,41], further validating the applicability of technology acceptance models in drone adoption. However, these factors have a more significant impact in rural areas, partly because rural communities have limited exposure to drone technology and greater concerns about potential technological failures [10,35]. In contrast, urban residents are more familiar with emerging technologies and are less sensitive to potential malfunctions. Perceived risk, particularly concerns about privacy and technology failures, is a critical factor influencing the acceptance of drone delivery services, especially in urban areas. Urban consumers tend to have heightened concerns regarding privacy and the potential for technological malfunctions, which significantly affects their willingness to adopt drones. This finding aligns with previous studies [8,13,31–33]. However, in rural areas, the impact of perceived risk is less pronounced. One possible explanation is that Chinese urban consumers, due to higher technological literacy, denser living environments, and stronger media exposure to privacy and safety incidents, place greater emphasis on risk mitigation, whereas rural consumers are more concerned with service accessibility and reliability, reflecting their more pragmatic and cautious attitudes toward adopting emerging technologies. This contrasts with the findings of a prior study conducted in the U.S [6]. In that study, the researchers conducted an online survey of U.S. consumers and applied regression analysis to the collected data, explicitly comparing results between urban and rural groups. They found that perceived risks, particularly related to privacy, were more significant for rural consumers. This discrepancy may stem from differences in developmental stages, regional characteristics, and cultural contexts between China and the U.S. Alternatively, the difference could arise from the broader range of risks considered in this study, which includes not only privacy concerns but also technological failures. Future research should further investigate how different forms of perceived risk—such as privacy, technical reliability, and safety—interact with demographic characteristics (e.g., age, education) to shape acceptance in diverse rural contexts.

Health safety concerns also differ between urban and rural groups. Urban residents show a stronger focus on health safety, whereas these factors have less impact in rural areas. Urban consumers may perceive drones as offering health safety advantages, such as avoiding contact with infections or accelerating medical deliveries, and some city residents who experienced drone delivery during the COVID-19 pandemic may continue to view drones as an essential tool in emergency relief [36,42,45,46,67]. In contrast, drones were indeed applied to rural medical deliveries during the pandemic, but the usage was still limited compared to urban areas. Rural residents may be less aware of the potential advantages of drone delivery and may have lower concerns about health safety due to more pressing livelihood concerns and relatively underdeveloped living conditions. Moreover, policy support has a more significant impact in urban areas, likely reflecting the higher attention urban residents pay to policy changes. While rural and remote areas are often seen as favorable testing grounds for drone delivery [9], Chinese e-commerce companies, such as JD.com, have been actively piloting drone delivery in rural provinces [3], and the government generally supports rural drone initiatives [34]. However, due to the lower educational levels of rural residents and limited information dissemination channels, their attention to policy matters is comparatively lower, which limits the effectiveness of policy support and makes its impact on rural residents relatively less. Interestingly, social norms have a more significant impact on drone acceptance in urban areas, likely due to denser social networks and more frequent interactions in cities. In rural areas, where social interactions are fewer, the influence of social norms is weaker. This finding challenges the assumption that social norms universally influence technology adoption [2,67–69], highlighting regional differences that previous studies have rarely addressed in rural contexts. A plausible explanation is that rural communities rely more on traditional word-of-mouth and interpersonal trust than on broad social influence, reducing the measurable effect of social norms. Future studies should adopt mixed methods (e.g., surveys combined with ethnographic or interview-based approaches) to capture the subtle and context-dependent role of social norms in rural adoption.

Service performance—particularly delivery speed—has a significant impact on drone acceptance, consistent with previous research [7,13,35,36]. However, compared to urban areas, the influence of service performance is more significant in rural areas, which contrasts with a previous global survey [7] suggesting that urban consumers, who frequently use app-based delivery services, tend to focus more on the convenience drones bring. While rural consumers could also benefit from improved services, they may initially be more cautious due to their lack of familiarity with the concept. The difference in the findings may be due to the greater logistical challenges faced in rural China compared to developed countries, where rural consumers place more value on the practical benefits of drone delivery, such as faster and more efficient service. This divergence suggests that service performance is not a universal driver but one moderated by infrastructure disparities. Future research should therefore compare rural regions with different levels of logistical development to clarify under what conditions service performance exerts the strongest influence.

In China, significant disparities between urban and rural areas in terms of socio-economic conditions, infrastructure, and cultural context have a profound impact on technology adoption, particularly for innovations such as drone delivery services. Urban residents typically enjoy higher levels of education, greater technological exposure, and higher incomes, which lead to a more favorable disposition toward emerging technologies like drones. In contrast, rural areas face limitations in educational resources and information dissemination channels, resulting in lower familiarity with and more cautious acceptance of new technologies. This is especially true when it comes to perceived usefulness and concerns about technological failure. Additionally, infrastructure differences play a crucial role. Urban areas benefit from well-established logistics and communication networks that support the widespread use of drone delivery, whereas rural areas often suffer from underdeveloped infrastructure, including poor transportation and limited internet coverage. As a result, rural residents are more likely to appreciate the potential of drones in addressing logistical inefficiencies and overcoming infrastructure gaps, making service performance, such as delivery speed, a more significant factor in rural acceptance. Cultural factors also contribute to these differences. Urban areas, with their more diverse and dynamic social networks, facilitate the rapid spread of information about new technologies, leading to greater acceptance of innovations like drone delivery. In contrast, rural communities rely more on face-to-face interactions and traditional forms of communication, which can limit the spread of information and make them more skeptical of unfamiliar technologies. Social norms in urban environments, therefore, exert a stronger influence on technology adoption than in rural areas. These disparities highlight the need for tailored strategies when promoting drone delivery technologies.

Given these findings, it is clear that different strategies should be adopted for urban and rural areas to effectively promote drone delivery services.

For logistics companies, this study highlights the importance of adopting differentiated strategies for urban and rural markets to effectively promote drone delivery services. In urban settings, firms should proactively reduce perceived risks by directly addressing consumer concerns related to privacy violations, technical malfunctions, and potential cost implications. In the urban group, perceived risk (Beta = −0.129) had a significant negative impact on willingness to accept, suggesting that addressing privacy and technical concerns is essential to fostering acceptance. Moreover, leveraging social norms through word-of-mouth marketing, community outreach, and public demonstration initiatives—particularly in densely populated environments—can serve as an effective means of enhancing trust and encouraging behavioral adoption. This is particularly important as social norms (Beta = 0.116) also had a significant positive influence on willingness to accept in the urban group. In contrast, rural areas—where awareness and familiarity with drone services remain relatively low—require a more foundational approach. Logistics providers are advised to implement targeted educational campaigns to improve public understanding of drone operations. In the rural group, perceived usefulness (Beta = 0.150) and perceived ease of use (Beta = 0.166) had a significant positive impact on willingness to accept, which suggests that emphasizing the practical benefits of drone delivery could foster greater interest. Additionally, emphasizing the service-related advantages of drones, such as delivery speed, operational reliability, and affordability, can help dispel skepticism and foster interest. However, rural areas showed a more significant effect for service performance (Beta = 0.151), suggesting that highlighting the reliability of service may be crucial for rural consumers. Furthermore, collaboration with local stakeholders and institutions—including schools, clinics, and agricultural cooperatives—as well as the deployment of pilot programs and free trial services, can serve as tangible demonstrations of value, thereby strengthening public trust and promoting broader acceptance in underserved regions. This is particularly important given that health safety (Beta = 0.076, p = 0.090) had a marginal positive impact in the rural group, while policy support (Beta = 0.046, p = 0.306) and social norms (Beta = 0.053, p = 0.222) were not significant, indicating that foundational education and trust-building initiatives may be more effective than policy measures in the short term.

For policymakers, the study suggests that they should consider the differences in acceptance between urban and rural areas and implement targeted policies to promote drone delivery services in China. For urban areas, governments should provide policy support by reducing flight restrictions, offering tax incentives, and providing subsidies. These measures should be informed by the positive influence of perceived usefulness (Beta = 0.128) and perceived ease of use (Beta = 0.143) in urban settings, emphasizing the importance of convenience and utility for urban consumers. They should also improve relevant laws and regulations to ensure the safety and privacy of drone deliveries. For rural areas, there should be greater efforts to raise awareness of drone delivery services and related policy support, improving rural residents’ understanding and acceptance of drone services. Given the stronger impact of service performance (Beta = 0.151) in rural areas, policymakers should also focus on promoting the operational advantages of drones in these regions. These factors had a more substantial effect on willingness to accept drone delivery services in rural areas compared to urban areas.

Additionally, regarding environmental factors, both urban and rural areas show that environmental cognition (Beta = 0.143), UAV-environment cognition (Beta = 0.088) in urban areas, and environmental cognition (Beta = 0.151), UAV-environment cognition (Beta = 0.153) in rural areas all significantly impact the acceptance of drone delivery services. To support the adoption of eco-friendly drone technologies, policymakers should establish green logistics policies, such as tax incentives and subsidies for logistics companies that implement sustainable practices. Moreover, developing environmental impact assessments and standards will ensure that drone operations do not negatively affect the environment, particularly concerning noise and carbon emissions. Drone companies should also invest in green technology, such as low-noise, low-emission drones, to meet regulatory requirements and enhance competitiveness. Furthermore, collaboration with local governments and agricultural cooperatives can facilitate pilot programs, helping to promote green logistics services and strengthen brand image. In addition, companies can actively raise public environmental awareness by launching targeted communication campaigns, integrating sustainability messages into marketing strategies, and highlighting the environmental benefits of drone delivery (e.g., reduced carbon footprint compared to traditional trucks). Educational outreach through social media, community workshops, and partnerships with schools or NGOs can further reinforce positive perceptions of drone technologies as environmentally friendly solutions. These initiatives not only improve consumer knowledge but also foster a stronger sense of environmental responsibility, thereby encouraging greater acceptance of drone delivery.

It is important to note that the current dataset over-represents younger and more educated respondents, which may influence the observed acceptance patterns. If older populations or those with lower technological exposure, particularly in rural areas, were better represented, several differences might emerge. Older or less technologically literate consumers may show greater resistance to drone delivery due to unfamiliarity with digital services, stronger concerns about reliability, or limited trust in autonomous systems. This could reduce the overall predictive strength of perceived usefulness and ease of use while amplifying the role of perceived risk. In rural contexts, where older residents rely more heavily on traditional delivery channels, acceptance might hinge less on environmental or policy considerations and more on visible demonstrations of safety, reliability, and tangible service benefits. Consequently, future research should incorporate more balanced demographic samples to capture these perspectives and provide a more accurate representation of nationwide consumer attitudes toward drone delivery.

## 7. Conclusions

This study provides important theoretical and practical implications for drone delivery services. In terms of green research, drones, as an emerging delivery tool, demonstrate the potential to reduce carbon emissions and lower energy consumption, aligning with sustainability goals. By revealing the positive impact of environmental cognition and UAV-environmental cognition on consumer acceptance, this study emphasized the importance of raising public environmental consciousness, which enhances drone acceptance and contribute to reducing the environmental impact of traditional logistics methods, thereby driving the development and application of green technologies. Furthermore, this study clarified the differentiated determinants and acceptance pathways between urban and rural consumers, offering empirical evidence to inform targeted industry strategies and policy frameworks and facilitating the broader diffusion and sustainable integration of drone logistics. Notably, by employing ridge regression analysis, the study achieved significant reductions in MAE, RMSE, and MAPE compared to traditional multiple linear regression, effectively addressing the issue of multicollinearity commonly encountered in conventional regression analyses and substantially improving the robustness and reliability of the empirical results.

The present study offers theoretical and managerial implications; however, it also has certain limitations that warrant careful consideration. First, some participants lacked prior experience with drones, particularly in rural areas where awareness of drone delivery is generally low. Despite providing respondents with informational materials and images of drone courier deliveries, their responses remained dependent on the information provided and their own imagination. Additionally, the over-representation of young, highly educated participants may have influenced the willingness to accept drone delivery, as younger and more educated individuals are generally more open to new technologies and innovations. This is particularly relevant because such respondents may have more positive perceptions of new technologies like drones, which could lead to an inflated willingness to adopt these services. In contrast, rural populations, especially those with less exposure to technology, may be more skeptical or cautious in their responses, even if they are exposed to information about drone deliveries. Second, the data samples were predominantly collected from market conditions in developing countries, which may limit the broader applicability of the study’s conclusions. Given the potential differences in attitudes and perceptions of drone delivery services among consumers in developed countries, caution is advised when generalizing these findings to other markets. Additionally, all variables in this study were measured using the same questionnaire, and no statistical test (e.g., Harman’s one-factor test) was conducted to control for potential common method variance (CMV). This may introduce bias into the results, and future research is recommended to explicitly assess and control for CMV risk.

The study recommends that future research include data collection from both urban and rural consumer segments in developed countries, allowing for comparative analysis with data from developing nations to gain a more comprehensive understanding of technology adoption across different economies. In addition, the use of more advanced data analysis methods—such as random forest models with SHAP interpretation, machine learning algorithms, deep learning approaches, and multilevel linear modeling—combined with big data mining and behavioral tracking experiments, is recommended to further elucidate the dynamic relationships among variables and real-world adoption processes. Moreover, instead of relying solely on survey-based models, future studies should incorporate real-world behavioral data obtained from pilot programs, large-scale trials, and operational platforms. Such data can be utilized to track actual consumer interactions with drone delivery services (e.g., frequency of use, delivery satisfaction, complaint records, and behavioral switching between drones and traditional couriers). By linking these behavioral indicators with demographic and attitudinal data, researchers can build more accurate prediction models that better capture adoption dynamics. In practice, this integration would allow machine learning models to be trained on both stated preferences (surveys) and revealed preferences (behavioral data), thereby improving predictive accuracy and reducing biases inherent in self-reported data. Finally, although this study presents a relatively comprehensive model, further investigation into additional potential influencing factors is necessary. Various factors, such as the value of the item, the nature of the goods, and cultural differences, may affect the willingness to accept drone delivery services and should be explored in future research.

## Supporting information

S1 Data Anonymized participant background information and questionnaire scale scores. (XLSX)

S2 Data Questionnaire Score Test Set. (XLSX)

S1 Table Questionnaire items and references. (DOCX)

S1 Text Information on drone courier delivery provided to respondents. (DOCX)
