# Supplementary material for: Examining the acceptance of drone delivery services among Chinese consumers: A perspective from urban and rural areas
Source: PLoS One. 2025 Sep 29;20(9):e0333422. doi: 10.1371/journal.pone.0333422 (PMC12478889; doi:10.1371/journal.pone.0333422)
Supplement: S1 Table — (DOCX) [file pone.0333422.s003.docx]

**S1 Table. Questionnaire items and references.**

| Variant | Subject | communalities (extracted values) | References |
| --- | --- | --- | --- |
| Environmental Cognition  (EC) | I am concerned about environmental issues.  I am also concerned about traffic congestion.  I am particularly worried about the environmental problems caused by motor vehicle emissions.  If everyone contributes, environmental problems can be significantly improved. | 0.744  0.732  0.770  0.689 | [[1](#_ENREF_1)] |
| UAV-  Environmental Cognition  (UAV-EC) | Drone courier delivery is more environmentally friendly.  Drone courier services help alleviate traffic congestion.  Powered primarily by batteries, drone courier deliveries can significantly reduce exhaust pollution. | 0.791  0.796  0.786 |  |
| Perceived  Usefulness  (PU) | Using drone courier deliveries can shorten my waiting time.  The use of drones for courier deliveries ensures that I receive my items promptly.  Drone courier services offer faster delivery times.  Overall, I find drone courier delivery to be highly useful. | 0.781  0.798  0.784  0.782 | [[2](#_ENREF_2),[3](#_ENREF_3)] |
| Perceived Ease Of Use  (PEOU) | Courier delivery with drones is straightforward.  No complex procedures are required to receive drone-delivered packages.  With drone courier deliveries, I don’t have to worry about operational difficulties.  Overall, I find drone courier delivery easy to use. | 0.706  0.757  0.754  0.733 | [[2](#_ENREF_2),[3](#_ENREF_3)] |
| Perceived Risk  (PR) | I am concerned about potential privacy breaches when using drone courier services.  I worry about the possibility of drones making errors in delivering packages.  I am also concerned about the risk of unreasonable or fraudulent charges when using drone courier services. | 0.808  0.790  0.822 | [[4](#_ENREF_4)] |
| Health Safety (HS) | Drone courier deliveries can minimize human contact and reduce the risk of disease transmission.  Drone courier services can respond to health emergencies and help protect public health.  Drone courier delivery can enhance the speed of emergency medical supply transport, safeguarding people's health.  Drone courier delivery can reduce ground traffic and help prevent traffic accidents. | 0.770  0.776  0.800  0.758 |  |
| Policy Support  (PS) | The more the country promotes drone courier delivery, the more willing I am to accept it.  The stronger the policy support, the greater my willingness to accept drone courier deliveries.  The better the insurance coverage, the more willing I am to accept drone courier services.  I would be more willing to accept drone courier deliveries if there were policies subsidizing delivery costs. | 0.752  0.805  0.738  0.719 |  |

**S1 Table. Cont.**

| Variant | Subject | communalities (extracted values) | | References | |
| --- | --- | --- | --- | --- | --- |
| Social Norm  (SN) | The more people who are willing to accept drone courier deliveries, the more inclined I am to accept them as well.  If drone courier deliveries are widely accepted, I would be willing to adopt them too.  I would accept drone courier deliveries in order to align with societal norms. | 0.772  0.762  0.759 | | [[5](#_ENREF_5)] | |
| Service  Performance  (SP) | Distribution costs  Delivery speed  Weight-carrying capacity  Technological maturity  Adaptability to severe weather conditions | 0.676  0.731  0.693  0.754  0.697 | [[6](#_ENREF_6)] | |  |
| Willingness  Accept  (WA) | I am willing to use drone courier delivery.  I am open to the idea of drone courier deliveries.  I would likely accept a drone courier delivery.  I would recommend drone courier delivery to my relatives and friends. | 0.782  0.786  0.735  0.782 | [[7](#_ENREF_7)] | |  |

Note: Communalities (extracted values) represent the proportion of variance in each item explained by the common factor(s). Values ≥0.50 indicate good representation of the item, while values <0.40 suggest weak representation and potential exclusion.

**References**

1. Schahn J, Holzer E. Studies of individual environmental concern: The role of knowledge, gender, and background variables. Environment and behavior. 1990;22(6):767-786.

2. Davis FD. Perceived usefulness, perceived ease of use, and user acceptance of information technology. MIS quarterly. 1989;13:319-340.

3. Venkatesh V, Davis FD. A model of the antecedents of perceived ease of use: Development and test. Decision sciences. 1996;27(3):451-481.

4. Featherman MS, Pavlou PA. Predicting e-services adoption: a perceived risk facets perspective. International journal of human-computer studies. 2003;59(4):451-474.

5. Barth M, Jugert P, Fritsche I. Still underdetected–Social norms and collective efficacy predict the acceptance of electric vehicles in Germany. Transportation research part F: traffic psychology and behaviour. 2016;37:64-77.

6. Ewing G, Sarigöllü E. Assessing consumer preferences for clean-fuel vehicles: A discrete choice experiment. Journal of public policy & marketing. 2000;19(1):106-118.

7. Hwang J, Lee J-S, Kim H. Perceived innovativeness of drone food delivery services and its impacts on attitude and behavioral intentions: The moderating role of gender and age. International Journal of Hospitality Management. 2019;81:94-103.
