# Supplementary material for: Examining the acceptance of drone delivery services among Chinese consumers: A perspective from urban and rural areas
Source: PLoS One. 2025 Sep 29;20(9):e0333422. doi: 10.1371/journal.pone.0333422 (PMC12478889; doi:10.1371/journal.pone.0333422)
Supplement: S1 Text — (DOCX) [file pone.0333422.s004.docx]

Information on drone courier delivery provided to respondents

【Introduction to drone courier delivery】

"Drone Courier Delivery" is an innovative delivery method that uses drones-small unmanned flying vehicles-to transport packages. This method of delivery is different from traditional ones, like trucks or delivery vans. Drones can carry items from one place to another much faster, especially in areas where roads might be blocked or hard to reach. Additionally, using drones can reduce delivery costs, as they don’t require drivers, and can be more efficient in certain situations, such as in emergencies or remote areas.

This innovative delivery method could help make deliveries faster, cheaper, and more environmentally friendly. However, like any new technology, it also has some challenges, such as safety concerns, regulations, and the ability to handle different weather conditions.

【Drone delivery pictures】


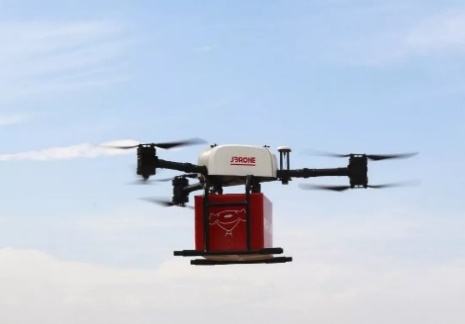


【Drone Delivery Video】

[
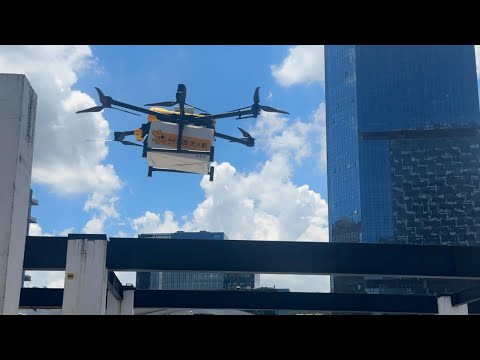
https://www.youtube.com/embed/_t_jP5Mrh44?feature=oembed](https://www.youtube.com/embed/_t_jP5Mrh44?feature=oembed)
